# Oral Fluorescein Angiography with Ultra-Wide-Field Scanning Laser Ophthalmoscopy in Pediatric Patients Precis: Oral Fluorescein Angiography in Children

**DOI:** 10.3390/jcm11185421

**Published:** 2022-09-15

**Authors:** Zhaoxin Jiang, Limei Sun, Aohan Hou, Ting Zhang, Yanting Lai, Li Huang, Xiaoyan Ding

**Affiliations:** State Key Laboratory of Ophthalmology, Guangdong Provincial Key Laboratory of Ophthalmology and Visual Science, Zhongshan Ophthalmic Center, Sun Yat-sen University, Guangzhou 510060, China

**Keywords:** oral fluorescein angiography, intravenous, scanning laser ophthalmology, pediatric, image quality

## Abstract

**Aims:** To investigate the success rate of oral fluorescein angiography (oral FA) in children with ultrawide scanning laser ophthalmoscopy (SLO) system and whether it can provide images of sufficient quality compared with intravenous FA (IVFA). **Methods:** In this comparative case series study, a series of 40 consecutive pediatric patients of the age of 3–18 with retinal vascular diseases, in whom FA was needed for the diagnosis or treatment, were enrolled in this study. IVFA and oral FA were performed within one week and images were obtained with the SLO system. The image quality was scored blindly and compared based on: (I) visualization of the branch retinal vessel, (II) the foveal avascular zone (FAZ), and (III) clinically important findings, such as the presence of microaneurysms, neovascularization, leakage, or significant nonperfusion. All these were scored using a three-point scale. **Results:** In preschoolers (three to six years), all 19 children complete oral FA (100%), while only 7 (36.84%) complete IVFA (*p* < 0.0001). With the SLO system, the branch retinal vessels were well visualized both in oral and IV FA (all images were two scores). The visualization of FAZ was similar between oral and IV FA (*p* = 0.8972). The clinically important findings were well visualized in both groups (*p* > 0.9999). The overall image quality was similar between the two groups (*p* = 0.2500). **Conclusion:** Oral FA is more acceptable to preschoolers than IVFA owing to the needle-free procedure. With the SLO system, oral FA provided high-quality angiograms similar to IVFA. Oral FA is an effective alternative to IVFA and may be considered the first option for FA in pediatric patients, especially in preschoolers.

## 1. Introduction

Fluorescein angiography (FA) has been routinely used in clinical practice to analyze retinal pathologies, by identifying the anatomical location and pattern of vascular leakage and is widely accepted as the gold standard in many diseases [1,2,3]. For a long period, FA is conventionally performed using an intravenous (IV) injection of sodium fluorescein. However, intravenously administered sodium fluorescein may cause several side effects, ranging from mild nausea to severe reactions such as anaphylactic shock, which were well documented [4,5,6]. Besides, IVFA is less tolerated by children and may have to be performed under anesthesia.

Oral FA has been proposed as an alternative to IVFA due to several advantages [7,8,9]. First, the incidence of a severe allergy to sodium fluorescein is far less with oral FA than with IVFA [5,10]. Hara, et al. explored the safety of oral FA in 1787 patients; only 31 patients (1.7%) experienced minimal itching or nausea, and no anaphylactic or other severe adverse effects were observed [11]. Besides, oral FA can be performed in patients who have a fear of needles and thus, can improve patient acceptance and cooperation. Third, risks associated with needle injection are also eliminated, which is useful when treating patients with the blood-borne disease. However, there is a lack of studies comparing the success rate between oral FA and IVFA.

Previously, the wide usage of oral FA was limited by poor image quality. Kelley et al. reported the first case of oral FA, which produced inferior quality images with a conventional fundus camera [11,12]. With the rapid development of scanning laser ophthalmoscopy (SLO), acquiring good-quality oral FA images of most retinal pathologies has been possible [13,14,15]. However, studies comparing the quality of angiograms simultaneously between oral FA and IVFA in pediatric patients are still lacking.

Therefore, the aim of this study was to investigate the success rate of oral FA in children and whether it can provide images of sufficient quality compared with IVFA.

## 2. Methods

This study was performed according to the tenets of the Declaration of Helsinki and was approved by the Medical Ethics Committee of Zhongshan Ophthalmic Center, Sun Yat-sen University (2014MEKY048). Informed consent was obtained from guardians of all included children who were under 18 years of age.

## 3. Procedures

Recruit criteria included: Pediatric patients of the age of 3–18 with retinal vascular diseases, in whom FA was needed for the diagnosis or treatment. A total of 40 patients (aged 3–16 years) were enrolled in this study, including 19 preschoolers (3–6 years), 19 school children (7–12 years), and 2 adolescents, between January 2021 and December 2021. All parent guardians agreed to the oral and IV procedure after explanation. A comprehensive ophthalmic examination, including best-corrected visual acuity (BCVA) assessment, intraocular pressure (IOP) measurement, slit-lamp biomicroscopy, and ultra-widefield scanning laser ophthalmoscopy (UWF-SLO, Optos California; Optos, PLC, Dunfermline, Scotland; equipped with Optos V2 Vantage review Software), were performed in each child. The exclusion criteria included: (1) Patients with a history of allergy to sodium fluorescein or a severe reaction to any allergen; (2) Patients with moderate-severe asthma, renal failure, or significant cardiac disease.

Oral FA and IV FA was performed consequently within 1-week. Retinal imaging of both IVFA and oral FA were performed by UWF-SLO. According to the manufacturer’s suggestion, allergy skin tests were performed before initiating IVFA. The dose of sodium fluorescein required to generate the optimal oral angiogram has been suggested to be 20–30 mg/kg [8,11]. In the current study, the sodium fluorescein, 25 mg/kg body weight mixed with 20 mL of water (1.25 mg/mL sodium fluorescein per kilogram of body weight), was administered orally to all patients. The patients were instructed to ingest the mixture in a gulp. The imaging system timer was started after the patient had consumed the full dose of sodium fluorescein orally. Pupil dilation was achieved by applying tropicamide (1%) before imaging was performed. UWF-SLO was used for noncontact high-resolution retinal angiography. The images were obtained every 15 s until the late arteriovenous phase was reached, and then every 30 s until the late phase was reached.

## 4. Image Analysis

For the quantitative evaluation of oral UWF-FA, Images were anonymized and saved as high-quality image files. The images were randomized and graded for quality by two experienced retina specialists (ZJ and LS). To reduce the bias, only late phase images were used for evaluation, and the transit times were not provided to the graders. To score the images, 3 parameters were used, which were modified from previous reports (I) visualization of the branch retinal vessel, (II) the foveal avascular zone (FAZ), and (III) clinically important findings, such as the presence of microaneurysms, neovascularization, leakage, or presence of nonperfusion area [5,13,16].

**Figure 1:**
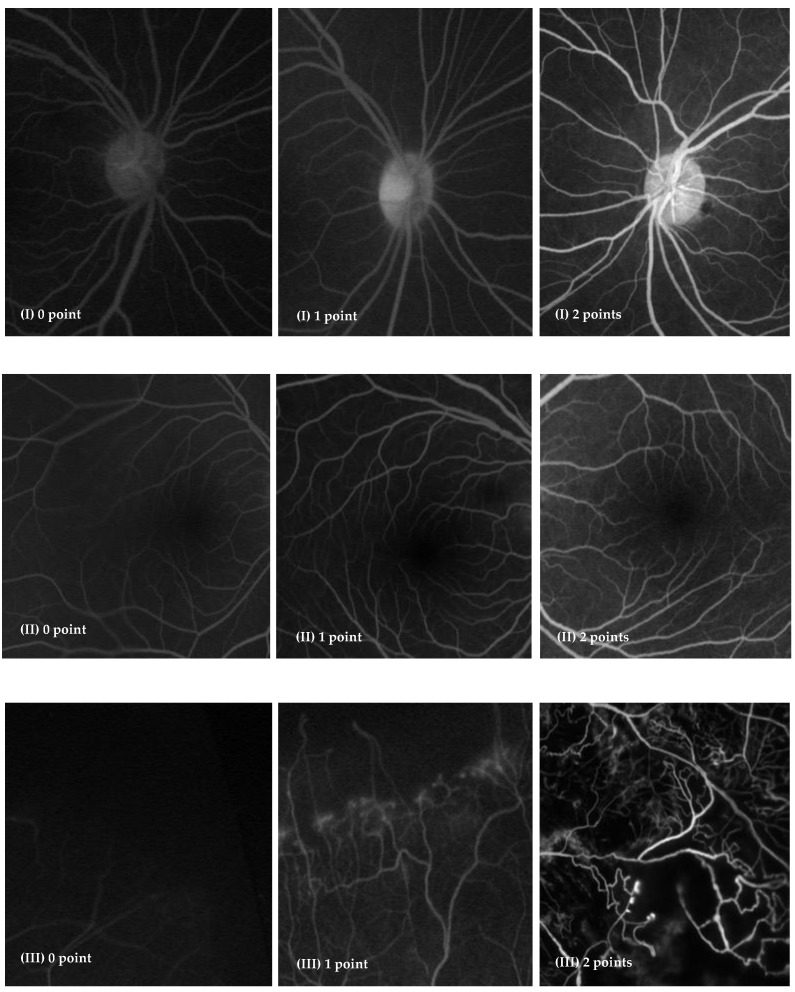
Representative images of each score regarding parameters I, II and III.

All these parameters were scored using a three-point scale as follows: (I) 0 points: only the first-order branches are seen, 1 point: second-order branches are seen, and 2 points: third-order branches are seen; (II) 0 points: not possible to judge the FAZ, 1 point: the FAZ is seen but not clearly, and 2 points: the FAZ is seen clearly and intact; and (III) 0 points: impossible to judge, 1 point: seen but not clearly, and 2 points: seen clearly. Representative images with scores of each of these parameters are shown in Figure 1.

## 5. Statistical Analysis

All data were collected in an electronic database and crosschecked for errors. For all parameters, intra- and interobserver agreements were calculated using the kappa statistic. The interpretation of kappa values was as follows: 0–0.20, slight agreement; 0.21–0.40, fair agreement; 0.41–0.60, moderate agreement; 0.61–0.80, substantial agreement; and greater than 0.81, almost perfect agreement. Statistical analysis included the non-parametric Wilcoxon test (paired *t*-test) and was performed using SPSS (version 16.0; SPSS, Chicago, IL, USA). Statistical significance was set at *p* < 0.05.

## 6. Results

### 6.1. Success Rate of Image Obtaining

There were 40/40 patients who completed oral FA, but 16 failed to complete IV FA due to needle fear (14 patients) and positive allergic skin tests to fluorescein (2 patients). Thus, the general success rate was significantly higher with oral FA than with IVFA (100% versus 60%, *p* < 0.0001). Considering the age, all 19 preschoolers completed oral FA, while only 7 of them completed IVFA (successful rate: 100% versus 36.84%, *p* < 0.0001). In school children, there was no significant difference in the success rate between oral and IVFA groups (successful rate: 100% versus 78.94%, *p* = 0.1050).

Thus, there were 24 patients who received both oral FA and IVFA in this study, including 8 girls (33.33%) and 16 boys (66.67%). The median age of these 24 patients was 8.08 (range 4–16) years, and the median body weight was 27.29 (range 15–54) kg. For the etiology, 11 cases were with familial exudative vitreoretinopathy (FEVR), 5 cases with Coats’ disease, 4 with chronic uveitis, 3 with ocular toxocariasis (OT), and 1 with high myopia (Table 1).

### 6.2. Image Quality Analysis

The median time for dye entry into the retinal circulation after fluorescein ingestion was 5.2 (range 4.5–6.3) min. The median time to optimal angiogram after ingestion was 10.5 (range 9.1–11.4) min, and the late-phase frame was visible at 15.7 (range 14.4–16.8) min.

**Figure 2:**
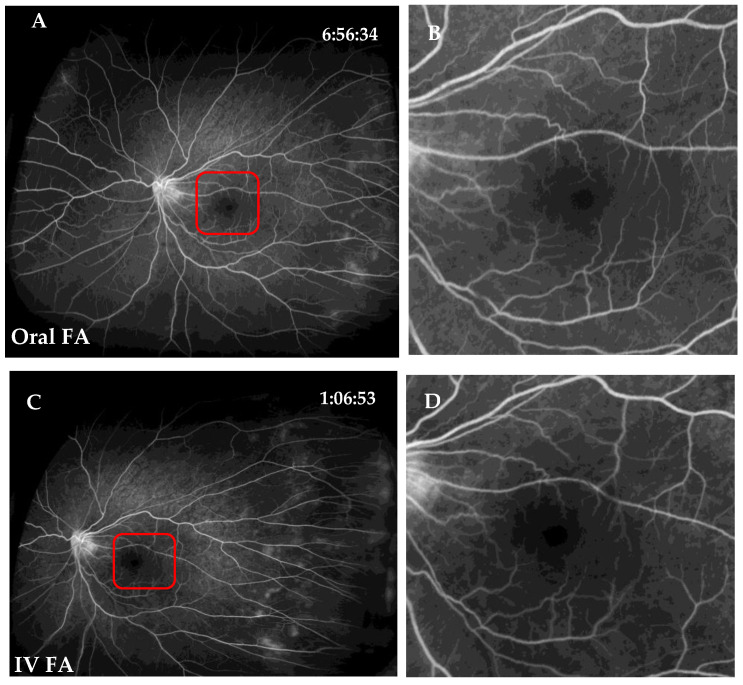

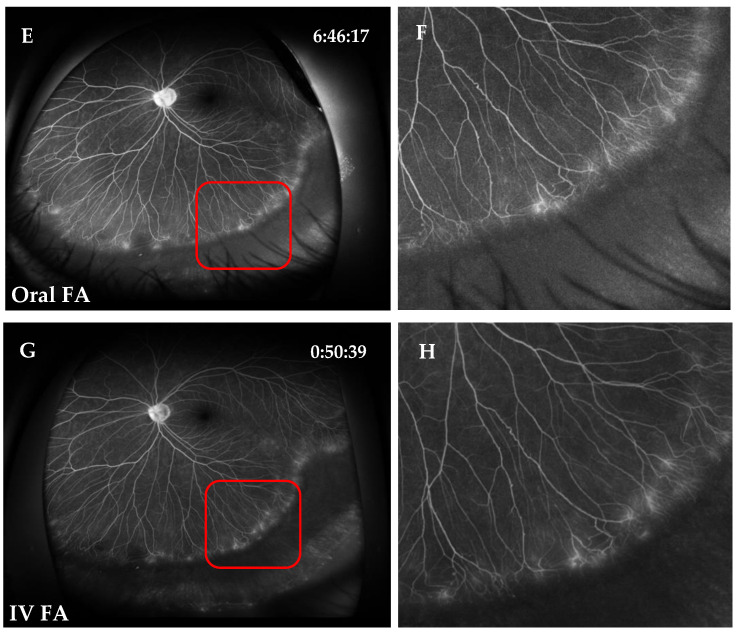
Representative images of the foveal avascular zone (FAZ) and non-perfusion area of Oral FA and IVFA. (**A**,**B**) Oral FA image of FAZ. (**C**,**D**) IVFA image of FAZ. (**E**,**F**) Oral FA image of non-perfusion area. (**G**,**H**) IVFA image of non-perfusion area. FA, fluorescein angiography; IVFA, intravenous fluorescein angiography.

**Figure 3:**
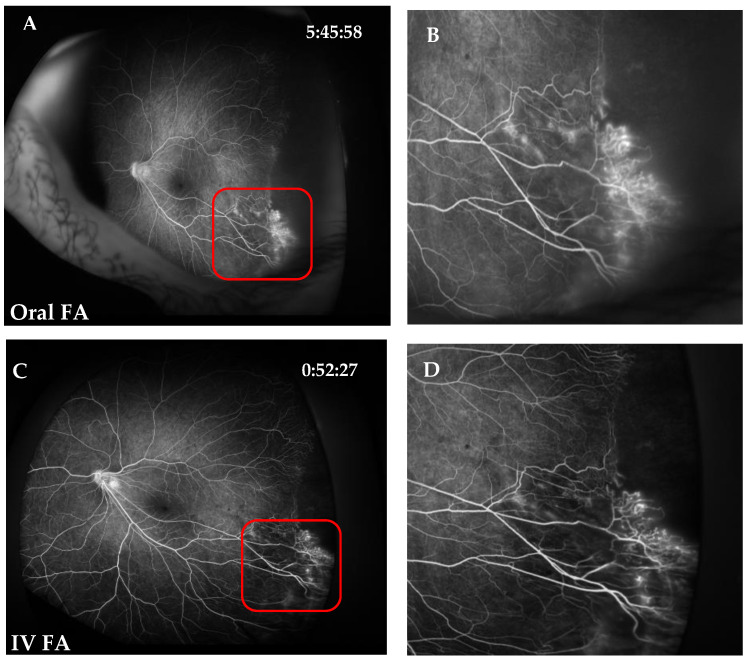

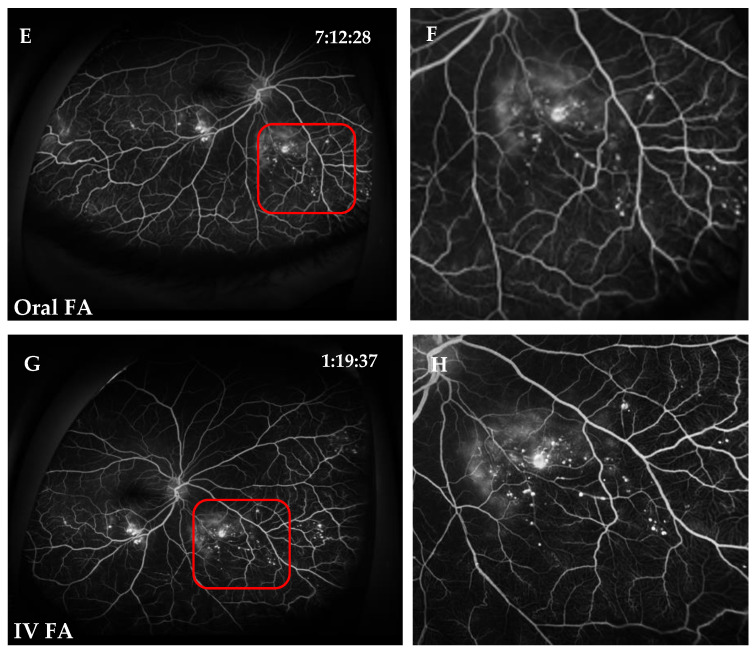
Representative images of the significantly nonperfused area, neovascularization, leakage, and microaneurysms of Oral FA and IVFA. (**A**,**B**) Oral FA image. (**C**,**D**) IVFA image. The nonperfused area with leaking telangiectatic vessels in the temporal peripheral retina is clearly visible in both oral FA and IVFA images. (**E**,**F**) Oral FA image of microaneurysms. (**G**,**H**) IVFA image of microaneurysms.

The kappa values of intra- and interobserver agreement were 1.0 for all parameters. Regarding parameter I scoring, central and branch retinal vessels were visualized well in both oral FA and IVFA images (score, 2) of all patients. Regarding parameter II scoring, the FAZ could be clearly seen in most eyes, except in 4 eyes with retinal folds or neovascularization in the macula. Visualization of the FAZ was also similar between oral FA and IVFA groups (*p* = 0.8972, Figure 2). Regarding parameter III scoring, the clinically important findings were visualized well in both groups (*p* > 0.9999, Figure 2 and Figure 3). For instance, the nonperfusion area with leaking telangiectatic vessels in the temporal peripheral retina was clearly visible in both oral FA and IVFA images. At last, the overall image quality was similar between the two groups (*p* = 0.2500, Table 1 and Table 2).

### 6.3. Adverse Events

Prior to IVFA, allergy skin testing through intradermal injection was performed to diagnose allergic reactions to fluorescein. Two patients showed positive skin responses and did not undergo IVFA. There was no adverse event during or after oral FA or IVFA.

## 7. Discussion

FA is one of the most important techniques for diagnosing retinal vascular diseases. However, the risk of severe allergy during IV injection of sodium fluorescein is a great concern for clinicians. Oral FA greatly reduces this incidence; however, its wide usage has been limited by the poor quality of images. Utilizing the UWF-SLO technique and an appropriate dose of sodium fluorescein, this study demonstrated that oral FA could generate high-quality images similar to IVFA, and could be a safe and convenient alternative to IVFA, especially in pediatric patients who might be uncooperative during IV injection and have difficulty in concentrating during image capture.

Generally, the oral administration route is much more accepted than IV in children. However, there is a lack of studies comparing the success rate between oral FA and IVFA. Hara et al. recruited 1787 patients who completed oral FA, and 266 of them received additional IVFA [11]. However, successful rates were not reported. Yamao et al. showed that 1 of 17 patients denied oral FA because of the bitter flavor of fluorescein sodium [8]. In the current study, we suggest there are at least two reasons contributing to the high success rate. The first is that oral FA is needle free. Taddio et al. showed that fear of needles was scored as strong in 68% of children aged 6–8 years [17]. Needle phobia, an anxiety disorder, could be diagnosed in 19% of children aged 4–6 years [18]. In addition, repeated IVs during follow up may cause severe distress in chronically ill children [19]. In current the study, the success rate was compared, and oral FA was much more acceptable by children. The second reason is a less allergic reaction in oral FFA. Adverse reaction to IVFA has an estimated incidence of 5%, with 0.05% considered severe [20]. As to oral FA, Hara et al. showed that 31 of 1787 patients (1.7%) experienced minimal discomfort [11]. In the current study, 2 out of 26 (7.69%) patients showed positive skin responses in skin testing prior to IVFA, while no allergy was reported in the oral FA group.

The image quality of oral FA improved well with the rapid development of SLO. Oral FA was first performed by Kelley et al. in 1979 [12]. However, studies that used a conventional fundus camera concluded that oral angiographic images were useful only in cases of central serous retinopathy or retinal pigment epithelial detachments [7,21,22]. The development of ophthalmic imaging techniques such as SLO has improved the detection of fundal abnormalities. Squirrell et al. evaluated the performance of oral FA with confocal SLO in the assessment of diabetic retinopathy and revealed that high-quality angiograms could be obtained in cases of maculopathy and peripheral nonperfusion [13,23]. Yamao et al. showed that oral FA with UWF-SLO was effective in evaluating clinically important factors such as FAZ visualization and neovascularization in pediatric patients [8]. In the current study, performances of oral FA and IVFA were compared in pediatric patients, and both techniques generated similar high-quality images.

Images quality of oral FA is greatly affected by indigestion of appropriate dose of fluorescein in a short time. In the current study, the amount of sodium fluorescein was 25 mg/kg of body weight, which was the same according to previous reports. However, it was mixed with 20 mL of water, making the concentration higher than that of previous reports, in which it was mixed with 30 mL or 100 mL of juice [24,25]. First, high concentration improves the image quality of oral FA. Faint images reported in studies were partly ascribed to a low concentration of fluorescein sodium, while all images were considered high quality in the current study. Second, low volume facilitates a fast indigestion. Ingested the mixture as a gulp, the circulation schedule was similar among patients and the examination duration was shortened. In the current study, the arteriovenous phase was usually reached at 5 min among patients. The late phase was reached as fast as 15 min, which was short than 30 or 60 min in previous studies [8,26]. The disadvantage is that an increased concentration of sodium fluorescein may increase the incidences of nausea and vomiting.

Based on similar image quality, oral FA has several advantages over IVFA. One of the main advantages is the reduction in complications associated with IVFA. IVFA has a moderate risk of adverse effects, which are rarely serious, while oral FA has shown no significant adverse effects in large cohorts [11,27,28]. Second, it is needle free and more acceptable to children. Traditionally, IVFA has been performed in adults and older children primarily because it requires a considerable level of patient cooperation, owing to the need for IV access and appropriate positioning for image capture [29,30,31]. In the current study, only 7 preschoolers (7/19, 36.84%) completed IV FA but all of them completed oral FA. Therefore, oral FA may be recommended as the first choice in pediatric patients, especially preschoolers.

On the other hand, oral FA had several disadvantages compared with IVFA. For instance, in oral FA, it was difficult to distinguish the classic FA phases, such as the arterial and arteriovenous phases, as clearly as IVFA. This was probably due to slower accumulation of the sodium fluorescein in oral FA than that in IVFA. Second, oral FA may not be suitable for all pediatric patients, including newborn and overweight children. Thus, we recommended that oral FA is not likely to completely replace IV FA, but may be an effective alternative for IVFA in pediatric patients.

Our results must be considered in light of several limitations. First, the spectrum of diseases was not broad enough and limited to FEVR, Coats’ disease and chronic uveitis. Patients with retinopathy of prematurity (ROP), one common and important pediatric retinal vascular disease, were not recruited in this study. Recent studies showed that UWF imaging provides a high-resolution panoramic view of the retina in infants with ROP [3,32]. Second, it is impossible to evaluate the safety of oral FA based on our results since the number of cases included in this study was small. No severe adverse events have been reported after oral FA in large cohorts [11]. Nonetheless, in a moderately large population, the current study showed oral FA as an effective alternative to IVFA, which is worth confirming in future studies.

## 8. Conclusions

Oral FA is more acceptable to preschoolers than IVFA owing to the needle-free procedure. With the SLO system, oral FA provided high-quality angiograms similar to IVFA. Oral FA is an effective alternative to IVFA and may be considered the first option for FA in pediatric patients, especially in preschoolers. Further investigations with a larger sample size from different cohorts is warranted to confirm our observations.

## Figures and Tables

**Table 1:** Demographic Characteristics and Image Sores of 24 Pediatric cases.

Case No.	Sex	Age Rangs (Years)	Weight (kg)	Diagnosis	Eyes	Images Score of Oral FFA (OD/OS)	Images Score of IV FFA (OD/OS)	*p* Value of Total Score
1	Male	<10	20	FEVR	OU	6/6	6/6	0.2500
2	Male	<10	22	HM	OS	Normal/6	Normal/6	
3	Male	10 s	39	FEVR	OU	6/6	6/6	
4	Male	10 s	31	FEVR	OU	6/6	6/6	
5	Male	<10	18	FEVR	OU	6/6	6/6	
6	Male	<10	22	FEVR	OU	6/6	6/6	
7	Female	10 s	31	OT	OS	Normal/5	Normal/6	
8	Male	<10	21	Coat’s	OS	Normal/6	Normal/6	
9	Male	<10	20	FEVR	OS	Normal/4	Normal/5	
10	Female	10 s	33	Coat’s	OD	6/Normal	6/Normal	
11	Male	<10	23	Coat’s	OS	Normal/5	Normal/6	
12	Male	10 s	39	OT	OS	Normal/4	Normal/4	
13	Male	10 s	54	Uveitis	OS	6/6	6/6	
14	Female	<10	27	FEVR	OU	6/6	6/6	
15	Female	10 s	50	Uveitis	OD	6/Normal	6/Normal	
16	Female	<10	17	Uveitis	OU	6/6	6/6	
17	Male	<10	32	FEVR	OU	4/6	4/6	
18	Male	<10	23	Coat’s	OS	Normal/6	Normal/6	
19	Female	<10	21	OT	OS	Normal/6	Normal/6	
20	Male	<10	24	Coat’s	OS	Normal/4	Normal/4	
21	Male	<10	30	FEVR	OS	6/4	6/4	
22	Male	<10	27	FEVR	OU	6/6	6/6	
23	Female	<10	16	FEVR	OU	6/6	6/6	
24	Female	<10	15	Uveitis	OS	Normal/6	Normal/6	

FEVR: familial exudative vitreoretinopathy, OT: ocular toxocariasis; HM: High myopia.

**Table 2:** Detection rates of retinal vascular characteristics for oral FA and IVFA.

Characteristics	Oral FA (n, %)	IVFA (n, %)	*p* Value
I. Visualization of branch retinal vessels visualization	36(100%)	36 (100%)	1.000
II. Visualization of foveal avascular zone	32 (100%)	32 (100%)	
III. Identification of clinically important findings	28 (100%)	28 (100%)	
Microaneurysms	4 (100%)	4 (100%)	
Neovascularization	4 (100%)	4 (100%)	
Leakage	9 (100%)	9 (100%)	
Significant nonperfusion	11 (100%)	11 (100%)	

FA: fluorescein angiography; IVFA: intravenous fluorescein angiography.

## Data Availability

All the data used to support the findings of this study are included within the article and are available from corresponding author by a reasonable request.
